# Composite Scaffold of Poly(Vinyl Alcohol) and Interfacial Polyelectrolyte Complexation Fibers for Controlled Biomolecule Delivery

**DOI:** 10.3389/fbioe.2015.00003

**Published:** 2015-02-03

**Authors:** Marie Francene A. Cutiongco, Royden K. T. Choo, Nathaniel J. X. Shen, Bryan M. X. Chua, Ervi Sju, Amanda W. L. Choo, Catherine Le Visage, Evelyn K. F. Yim

**Affiliations:** ^1^Department of Biomedical Engineering, National University of Singapore, Singapore, Singapore; ^2^INSERM, U698 Cardiovascular Bioengineering, Paris, France; ^3^INSERM, U791 Center for OsteoArticular and Dental Tissue Engineering, Nantes, France; ^4^Mechanobiology Institute, National University of Singapore, Singapore, Singapore; ^5^Department of Surgery, Yong Loo Lin School of Medicine, National University of Singapore, Singapore, Singapore

**Keywords:** controlled release, hydrogel, interfacial polyelectrolyte, permeability, angiogenesis

## Abstract

Controlled delivery of hydrophilic proteins is an important therapeutic strategy. However, widely used methods for protein delivery suffer from low incorporation efficiency and loss of bioactivity. The versatile interfacial polyelectrolyte complexation (IPC) fibers have the capacity for precise spatiotemporal release and protection of protein, growth factor, and cell bioactivity. Yet its weak mechanical properties limit its application and translation into a viable clinical solution. To overcome this limitation, IPC fibers can be incorporated into polymeric scaffolds such as the biocompatible poly(vinyl alcohol) hydrogel (PVA). Therefore, we explored the use of a composite scaffold of PVA and IPC fibers for controlled biomolecule release. We first observed that the permeability of biomolecules through PVA films were dependent on molecular weight. Next, IPC fibers were incorporated in between layers of PVA to produce PVA–IPC composite scaffolds with different IPC fiber orientation. The composite scaffold demonstrated excellent mechanical properties and efficient biomolecule incorporation. The rate of biomolecule release from PVA–IPC composite grafts exhibited dependence on molecular weight, with lysozyme showing near-linear release for 1 month. Angiogenic factors were also incorporated into the PVA–IPC grafts, as a potential biomedical application of the composite graft. While vascular endothelial growth factor only showed a maximum cumulative release of 3%, the smaller PEGylated-QK peptide showed maximum release of 33%. Notably, the released angiogenic biomolecules induced endothelial cell activity thus indicating retention of bioactivity. We also observed lack of significant macrophage response against PVA–IPC grafts in a rabbit model. Showing permeability, mechanical strength, precise temporal growth factor release, and bioinertness, PVA–IPC fibers composite scaffolds are excellent scaffolds for controlled biomolecule delivery in soft tissue engineering.

## Introduction

Controlled delivery of hydrophilic, protein-based drugs is widely explored for treatment of various diseases. It is postulated that the sustained release of proteins to a targeted area is a more effective method of achieving therapeutically relevant protein concentrations and preventing pathological doses (Huang and Brazel, 2001). However, currently available protein delivery systems, usually in the form of micro- or nano-spheres, are plagued by low incorporation efficiency and loss of costly recombinant proteins, loss of bioactivity caused by processes involving organic chemicals and multi-step processes, and uncontrollable temporal release (King and Patrick, 2000; Patel et al., 2008; Rui et al., 2012; Simón-Yarza et al., 2013).

Interfacial polyelectrolyte complexation (IPC) fibers have the characteristics that are especially pertinent for controlled release of protein-based therapeutics such as growth factors. IPC fibers have been utilized for precise spatiotemporal delivery of various biomolecules such as small drugs (Liao et al., 2005), large protein growth factors (Cutiongco et al., 2014; Teo et al., 2014), or even cells (Yim et al., 2006). Formation of IPC fibers is a self-assembly process that occurs at the interface of two oppositely charged polyelectrolytes in aqueous solution (Wan et al., 2004). High incorporation efficiency of potentially any type of biomolecule is possible with its addition to the similarly charged polyelectrolyte (Cutiongco et al., 2014). Furthermore, the ambient and aqueous conditions used for IPC fibers fabrication is advantageous in retaining the bioactivity of incorporated biologics. The facile, single-step fabrication of IPC fibers bestows versatility, permitting different geometries, alignment, and combination with other tissue engineering scaffolds (Cutiongco et al., 2014). Though IPC fibers were found to be compatible with intramuscular implantation, it was found to have low tensile strength due to lack of crystallinity and chain alignment (Yim et al., 2006). Moreover, the small size of IPC fibers fail to provide bulk mechanical support for most tissue engineering applications.

To overcome these structural limitations, IPC fibers can be incorporated into polymeric materials to create composite scaffolds (Cutiongco et al., 2014; Teo et al., 2014). Poly(vinyl alcohol) (PVA) hydrogel is non-carcinogenic, non-immunogenic, and highly biocompatible synthetic polymer that is popular for biomedical applications (Chaouat et al., 2008; Jiang et al., 2010). The cross-linked hydrogel, allowing water imbibition while maintaining structural integrity, has found a variety of uses in medicine and therapy. For instance, the elastic mechanical property and transparency of PVA make it an attractive choice for potential biomedical applications such as artificial cornea (Latkany et al., 1997), heart valve stent (Wan et al., 2002), contact lens (Yang et al., 1981), wound dressing (Taylor, 1963), and as a scaffold for drug delivery (Nugent and Higginbotham, 2007). Like IPC fibers, the ease of fabrication and versatile application of PVA makes it an attractive scaffold of choice. We hypothesize that IPC fibers embedded in PVA hydrogel will provide a composite scaffold that has temporally controllable biomolecule release, good mechanical integrity, and biocompatibility.

In this study, we explored the use of a composite scaffold of IPC fiber and PVA hydrogel (PVA–IPC fiber composite) as a vehicle for the temporally controlled release of hydrophilic biomolecules. We characterized the permeability properties of PVA hydrogel using model molecules lysozyme and bovine serum albumin (BSA) and observed the size- and charge-dependent permeability of PVA to these biomolecules. To explore its potential application as a vascular graft with temporally controlled growth factor delivery, PVA–IPC composite grafts were created. Incorporation of molecules in PVA–IPC composite grafts ranged from 80 to 97% efficiency. PVA–IPC composite grafts showed structural integrity and mechanical properties matching the rabbit femoral artery. The release profiles for AcSDKP angiogenic peptide, insulin, PEGylated-QK peptide (PEG-QK) angiogenic factor, lysozyme, and vascular endothelial growth factor (VEGF) indicated that release from PVA–IPC grafts depended on molecular weight and hydrodynamic radius. The release of the angiogenic factors VEGF and PEG-QK from PVA–IPC composite grafts stimulated endothelial cell metabolism, indicating retention of growth factor bioactivity. *In vivo* implantation of PVA–IPC composite grafts with mechanical properties matching rabbit femoral artery did not show significant degradation after 28 days. In addition, macrophage infiltration and capsule formation was similar between PVA–IPC and PVA grafts, indicating similar bioinertness. Thus, we demonstrated the mechanical integrity, capability for temporal growth factor release and bioinertness of PVA–IPC fibers composite scaffolds, with a potential application as a vascular graft.

## Materials and Methods

### Preparation of PVA

A 10% aqueous solution of PVA (Sigma-Aldrich, 85–124 kDa, 87–89% hydrolyzed) was prepared by dissolving PVA in distilled deionized (DDI) water at 121°C for 20 min using an autoclave. After mixing to ensure homogeneity, the PVA solution was used immediately or stored at 4°C until further use. The aqueous solution of PVA (12 g) was cross-linked by the addition of 1000 μl of 15% (w/v) sodium trimetaphosphate (STMP) (Sigma-Aldrich) and 400 μl of 30% (w/v) sodium hydroxide. To create planar PVA films for permeability assay (see Testing PVA Permeability to Biomolecules), glass coverslips were dipped in cross-linking PVA solution and left to dry at 18°C for 3 days. A cylindrical mold with a uniform outer diameter was dipped four times into a cross-linking PVA solution, with an interval of 15 min at each dip. The scaffolds were dried at 18°C and 60–80% humidity conditions for 3 days. Afterwards, the PVA grafts were used for making PVA–IPC grafts (see Fabrication of PVA–IPC Grafts with Different IPC Fiber Orientation).

### Testing PVA permeability to biomolecules

To assess the permeability of the PVA film to biomolecules, a leak-proof diffusion chamber was set up. PVA planar films comprising of four layers were mounted using a commercially available disposable insert (CellCrown, Scaffdex, Finland) to get a final diffusion area of 80 mm^2^. The top permeability chamber was filled with 800 μl of 1.5 mg/ml aqueous solution of either lysozyme (Sigma-Aldrich) or BSA (Sinopharm Chemical Reagent, China). The bottom chamber (enclosed by the well plate) was filled with 800 μl of DDI water. The plate was incubated at 37°C and 20 μl sample volumes were drawn from both chambers at specific timepoints. Protein concentrations were quantified using a BCA assay kit (Pierce) and permeability coefficients were calculated as described by Gao et al. (2008).

### Encapsulation of biologics in PVA–IPC composite graft

**Figure 1:**
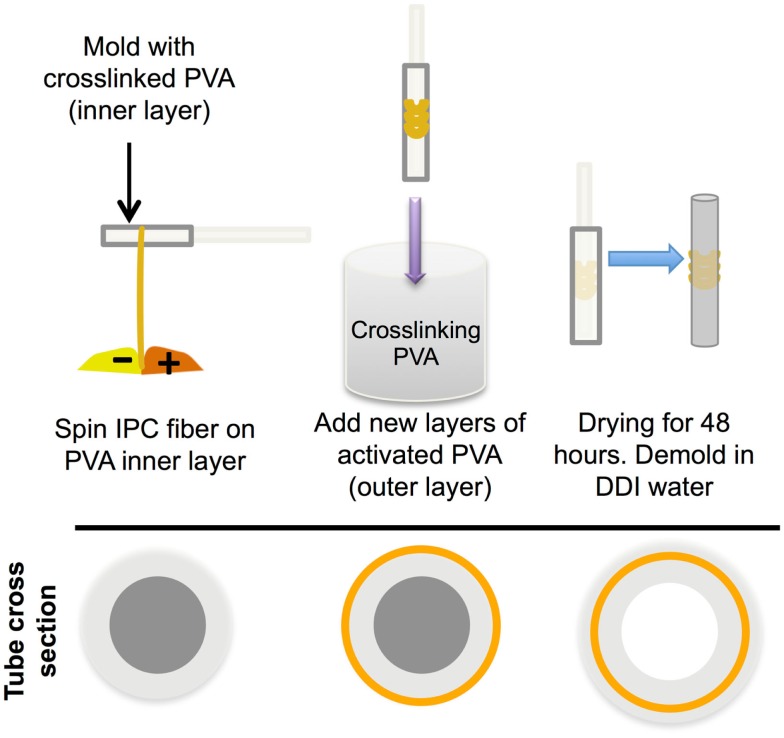
**Incorporation of IPC fibers to create a PVA–IPC fiber composite scaffold**. Schematic diagram of PVA–IPC composite scaffold fabrication method.

Purified chitosan (Low molecular weight, >75% acetylation, 20–300 cps; Sigma-Aldrich; 1% w/v in 0.15 M acetic acid), alginic acid (1% w/v in DDI water), and heparin (1% w/v in DDI water) were prepared, as described previously (Liao et al., 2005; Cutiongco et al., 2014). Alginate and heparin (9:1 ratio) were used for incorporation of VEGF in IPC fibers. The addition of heparin to the polyelectrolyte solution has been shown to attenuate the burst release of VEGF (Cutiongco et al., 2014). Chitosan and alginate or alginate-heparin solutions were drawn together to form IPC fiber through the use of a rotating mandrel at a constant rate of 10 mm/s. The IPC fiber was slowly drawn upward from the interface of two polyelectrolytes and the freshly drawn end was immediately stuck on the dry surface of a PVA film (see Fabrication of PVA–IPC Scaffolds with Different IPC Fiber Orientation), which was placed 2–2.5 cm above the polyelectrolyte solutions (Figure 1). Five types of biologics were encapsulated in the IPC fibers: AcSDKP peptide (Abbiotec, LLC), bovine insulin (Sigma-Aldrich), PEGylated-QK peptide (Supplementary Material), lysozyme, and VEGF (Life Technologies). The characteristics and total mass of each biomolecule incorporated into IPC fibers is summarized in Table 1. The total concentration of VEGF and PEG-QK incorporated was based on the minimum requirements for a sustained angiogenic response in rabbit hindlimb ischemia model (Hopkins et al., 1998). Total amount of PEG-QK incorporated was calculated from the molar equivalent of QK peptide necessary to simulate VEGF response (Santulli et al., 2009). To ensure incorporation during IPC fiber formation, negatively charged biologics were added to alginate while positively charged biologics were added to chitosan (Table 1). Upon IPC fiber termination, 500 μl of phosphate buffered saline (PBS) was used to collect the residue. The concentration of biologics in this solution was used to calculate incorporation efficiency. The ratios of chitosan:alginate used to incorporate each biomolecule is given in Table 2.

**Table 1:** **Properties of hydrophilic biomolecules used in this study**.

Biomolecule	Molecular weight (kDa)	Isoelectric point	Charge	Hydrodynamic radius (Å)	Amount (μg)
AcSDKP peptide	0.4	5.5	Negative	7^a^	1000
Insulin	5.73	5.3	Negative	13	111
PEG-QK	11.27	9.4	Positive	36.9^a^^,^^b^^,^^c^	27
Lysozyme	14.3	11.35	Positive	19.1	1200 (transwell) 350 (release)
VEGF	45.5	9.22	Positive	30.2	7.5
BSA	66.4	4.7	Negative	36.3	1200

*^a^Hydrodynamic radius of peptide with N residues predicted by formula 4.75N^0.29^ Å (Wilkins et al., 1999)*.

*^b^Hydrodynamic radius of 10 kDa PEG is 28 Å (Stiehl et al., 2013)*.

*^c^Hydrodynamic size of PEGylated peptide is the sum of the hydrodynamic radii of each component (Jevsevar and Kunstelj, 2012)*.

**Table 2:** **Incorporation efficiency of various hydrophilic biomolecules into PVA–IPC composite scaffolds**.

Biomolecule	Chitosan:Alginate:Heparin ratio	Efficiency (%)
AcSDKP peptide	1:1:0	96.67 ± 1.15
Insulin	1:1.7:0	91.74 ± 2.29
PEG-QK	1:1.3:0	75.1 ± 5.9
Lysozyme	1:1:0	78.7 ± 8.38
VEGF	1:1.1:0.1	96.41 ± 0.81

### Fabrication of PVA–IPC scaffolds with different IPC fiber orientation

Fully cross-linked PVA film with dimensions of 3.5 mm × 50 mm with 0.5 mm thickness was mounted on the rotating mandrel and used to collect IPC fibers. Immediately after drawing at the interface using fine-tipped forceps, the end of the IPC fiber was attached to the PVA film (Cutiongco et al., 2014). The IPC fibers were aligned either perpendicular or parallel to the long axis of the PVA film. Thereafter, the PVA–IPC constructs were dipped to add an additional six layers of cross-linking PVA solution (see Preparation of PVA).

### Fabrication of PVA–IPC grafts with different IPC fiber orientation

To fabricate PVA–IPC grafts, PVA cylindrical scaffolds with four layers were used (see Preparation of PVA). PVA–IPC composite grafts with circumferential IPC alignment (Circumferential grafts) were made by drawing IPC fibers onto a PVA cylindrical scaffold mounted on rotating mandrel. IPC fibers spanned the middle 0.5 cm section of a 1 cm long graft (Figure 1). AcSDKP, lysozyme, and VEGF were incorporated in these circumferential grafts. Biangular IPC fiber alignment on composite grafts (Biangular grafts) was achieved by oscillating the platform carrying the polyelectrolyte solution (8.57 mm/min) while PVA cylindrical scaffold was mounted on a rotating mandrel. IPC fibers spanned the whole length of the 1 cm long graft. Insulin and PEG-QK were incorporated in these Biangular grafts. Thereafter, IPC fibers mounted on a PVA tubular scaffold were allowed to dry at 4°C before dip-coating with four additional layers of cross-linking PVA. PVA–IPC grafts were removed from the tubular mold by washing in 10X PBS to minimize burst release of biomolecules. As a control for bioactivity assay (see Bioactivity Assay for Released Angiogenic Factors), standalone IPC fibers incorporated with biomolecules were fabricated.

### SEM analysis of PVA–IPC grafts

Cross-sections of grafts were air-dried overnight at ambient temperature. Dried grafts were platinum coated with an approximate thickness of 10 nm (JEOL-JFC 1600 auto-fine coater). Scanning electron microscope (SEM) JEOL-JSM 6010LV at high vacuum and 10 kV electrode voltage was used to visualize ultrastructure of PVA tubes.

### Mechanical characterization of PVA–IPC composite scaffolds

#### Wall thickness and internal diameter

Digital images of cross-sections of hydrated grafts were used to measure wall thickness (*n* = 7) and internal diameter (*n* = 5) using ImageJ 1.46j.

#### Uniaxial tensile test

Uniaxial testing (INSTRON 3345) was carried out on 2–3 cm length of hydrated scaffolds (*n* = 3). A 10-N load cell, cross-head speed of 10 mm/min, and gauge length of 1 cm were used. Samples were first subjected to a pre-load of 0.1 N prior to measurement. Planar PVA–IPC composite scaffolds were stretched until rupture to obtain maximum tensile strength.

#### Radial compliance test

PVA-IPC grafts (*n* = 6) were subjected to internal hydrostatic pressures of 80 and 120 mmHg. Images of grafts were taken at both pressures (Nikon SMZ745T stereomicroscope) and compliance was calculated by obtaining the percent change in the diameter of the PVA graft between the two pressures.

#### Burst pressure test

Closed-ended grafts (*n* = 3) were progressively filled with nitrogen gas. Burst pressure was recorded as the pressure at graft failure.

### Controlled release assay for biomolecules

Each PVA–IPC composite graft was placed in a 24-well plate with 500 μl of release medium. PVA–IPC grafts with AcSDKP, lysozyme, and insulin used PBS as release medium. Release medium for Circumferential grafts with VEGF (VEGF graft) was 0.5% BSA in endothelial basal medium (EBM, Lonza), which was then directly used in the bioactivity assay (see Bioactivity Assay for Released Angiogenic Factors). The amount of VEGF released at different time points was measured using human VEGF ELISA Kit (Life Technologies). Controlled release of Biangular grafts with PEG-QK (PEG-QK grafts) was performed simultaneously in two different release media. One set of samples was immersed in 500 μl of sterile PBS with 0.05% sodium azide, which was used to measure the cumulative release using PEG-ELISA, which contains the following: Anti-PEG IgM antibodies and anti-IgM horseradish peroxidase-conjugated antibodies (Academica Sinica) (Tsai et al., 2001). A second set of PEG-QK grafts was immersed in 500 μl of EBM, which were subsequently used for bioactivity assay (see Bioactivity Assay for Released Angiogenic Factors). Standalone IPC fiber samples incorporated with VEGF or PEG-QK were included as controls.

Cumulative release of the biomolecules was given as percentage of the total biomolecule mass incorporated. Apparent permeability coefficients were calculated according to Gao et al. (2008). The slope of the initial linear portion of the controlled release profile was used to calculate the permeability coefficient for each biomolecule.

### Bioactivity assay for released angiogenic factors

Human umbilical vein endothelial cells (HUVEC, Lonza, passages 4–5) were seeded on 96-well plate at 2500 cells/well and grown for 24 h in endothelial growth medium with 2MV bullet kit (EGM-2MV; Lonza). Cells were then serum-deprived for 24 h using EBM. To prevent extensive cell death, cells were afterwards cultured in 50 μl release solution supplemented with 50 μl of 2X concentrated EGM-2MV, resulting in 100 μl 1X EGM-2MV media with released VEGF or PEG-QK. After 48 h of incubation with release media, 10 μl of Alamar blue (Life Technologies) was added to each well to measure cell metabolic activity. After 4 h, absorbance of Alamar blue was measured at 570 and 600 nm (Tecan Infinite 200 PRO), as per manufacturer’s instructions. Cell metabolic activity was reported as percent Alamar blue reduction, which was normalized to the activity of cells grown in 0.5% BSA in EBM (negative control). HUVEC grown in EGM-2MV supplemented with 10 or 50 ng/ml VEGF was used as positive control. Each release medium sample was tested in triplicate.

### Implantation of PVA vascular graft in rabbit model

Graft implantation study was performed in accordance with approved guidelines of the Institutional Animal Care and Use Committee of the National University of Singapore. Rabbits (New Zealand White, Male, 3.5–4.0 kg) were sedated with ketamine (25 mg/kg) and medetomidine (0.02 mg/kg) and maintained on isoflurane (2–5%) plus O_2_ gas at 12 ml/min/kg. Enrofloxacin (5 mg/kg) and buprenorphine (0.04 mg/kg) were administered before surgery. After cut down and exposure of the left femoral artery, papaverine (1.43 mg/ml) was administered topically and intravascularly. Plain PVA (*n* = 3) or VEGF grafts (*n* = 3) with 1 cm length and 1 mm internal diameter were used. PVA grafts were anastomosed to left femoral artery using simple interrupted sutures (Ethilon, non-absorbable, 10-0). During this period, the femoral artery was occluded with hemostatic arterial clamps and heparin (100 IU/kg) was administered intravenously. In all cases, occlusive anastomosis time did not exceed 1.5 h. Ischemic subjects (positive control) were obtained through permanent ligation and resection of 4 mm length of the left femoral artery. Unoperated subjects (negative control) were also included. All subjects were treated with enrofloxacin (5 mg/kg) and buprenorphine (0.04 mg/kg) for 7 days post-operatively. No anticoagulants or antiplatelets were administered throughout the experiment. Subjects were kept for approximately 4 weeks.

### Histological analysis of PVA grafts

Rabbits were euthanized with sodium pentobarbital (150 mg/kg). Portions of the cranial abductor, semimembranous, and femoral bicep hindlimb muscle groups surrounding the graft or the resected femoral artery were harvested and fixed with 4% paraformaldehyde for 48 h. Tissues were then processed and embedded in Paraplast (Leica). Sections with 15 μm thickness were stained with routine hematoxylin and eosin (H&E). Assessment of macrophage infiltration was performed through detection of Mac-387 (Abcam) using standard immunohistochemical techniques (Frangogiannis et al., 2003). Ischemic and unoperated limbs were included as positive and negative controls, respectively.

### Statistical analysis

Data are presented as mean ± standard deviation. Student’s *t*-test was performed using GraphPad Prism v6.0 for statistical analysis of graft mechanical properties. All other statistical analyses were performed using ANOVA with Tukey’s *post hoc* test. Differences between means were considered statistically significant at *P* < 0.05.

## Results

### Permeability of biomolecules through PVA film

**Figure 2:**
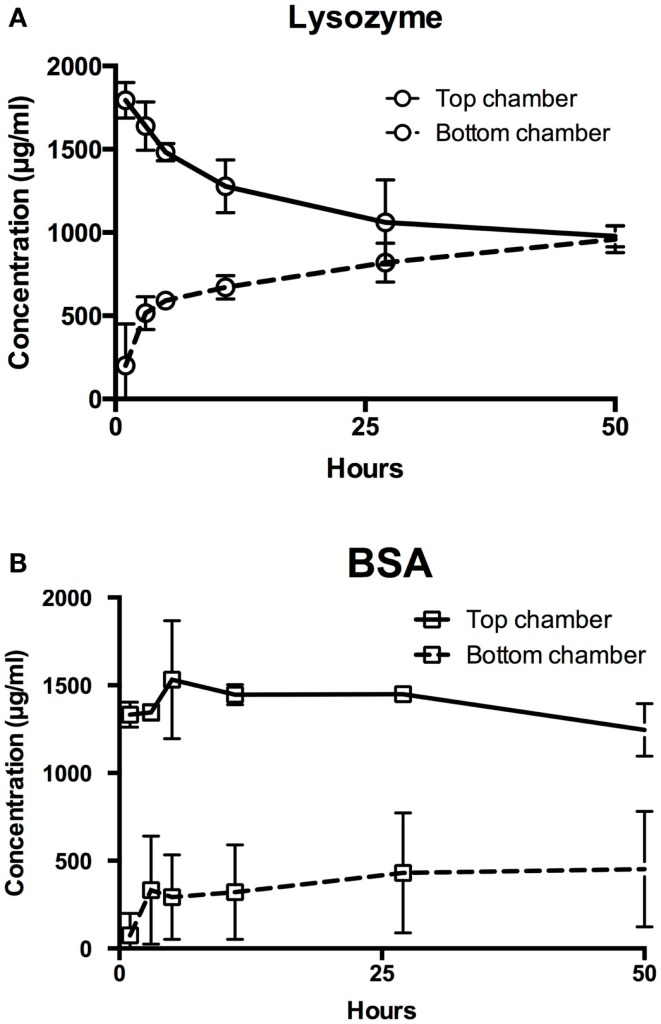
**Permeability of hydrophilic biomolecules through PVA**. Transwell assay was used to assess permeability of **(A)** lysozyme and **(B)** BSA through PVA film. Top chamber denotes chamber anterior to the transwell insert, where the biomolecules were added. Bottom chamber denotes the chamber enclosed by the tissue culture well plate and posterior to the transwell insert, where biomolecules diffuses through.

As a preliminary test, model molecules lysozyme and BSA were used to determine the permeability of PVA films to biomolecules (Figure 2). Lysozyme concentrations equilibrated between the top and bottom chambers within 24 h (Figure 2A). In contrast, the concentrations of BSA in both top and bottom chambers remained relatively stable over 48 h, demonstrating that BSA had minimal diffusion through the planar PVA film (Figure 2B). The calculated apparent permeability coefficients matched the observed relative permeabilities of the biomolecules through PVA, where higher permeability coefficient represents faster diffusion through PVA (Table 3).

**Table 3:** **Apparent permeability coefficients of biomolecules calculated from permeability experiment**.

Biomolecule	Apparent permeability coefficient (10^−7^ cm/s)
Lysozyme	32.98
BSA	9.723

### Fabrication of PVA–IPC composite films and grafts

**Figure 3:**
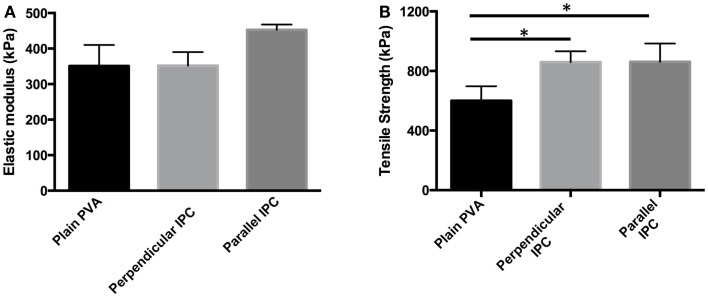
**Mechanical properties of PVA–IPC composite films with IPC fibers in different orientations**. **(A)** Elastic modulus and **(B)** maximum tensile strength of PVA–IPC composite films compared with plain PVA film. Orientation of IPC fibers are given with respect to the longitudinal axis of the PVA film and direction of strain. *Denotes statistical significance.

After showing permeability of PVA to two model molecules, we fabricated composite scaffolds that combined the controlled release capacity of IPC fibers with PVA. Planar PVA–IPC films were made to determine the effect of IPC fiber alignment with respect to strain direction on mechanical properties. We observed that elastic moduli of PVA–IPC composite films were statistically similar regardless of IPC fiber presence or orientation (Figure 3A), yet maximum tensile strength of each PVA–IPC film was significantly higher than the plain PVA (Figure 3B). However, no significant difference was observed between PVA–IPC with perpendicular IPC or parallel IPC. Since the parallel or perpendicular alignment of IPC fibers along the longitudinal axis had no significant differences in tensile strength, we fabricated PVA–IPC composite grafts with IPC fibers arranged along the circumferential axis of the tube. These PVA–IPC grafts were fabricated as a potential application of the composite scaffold in vascular tissue engineering.

**Figure 4:**
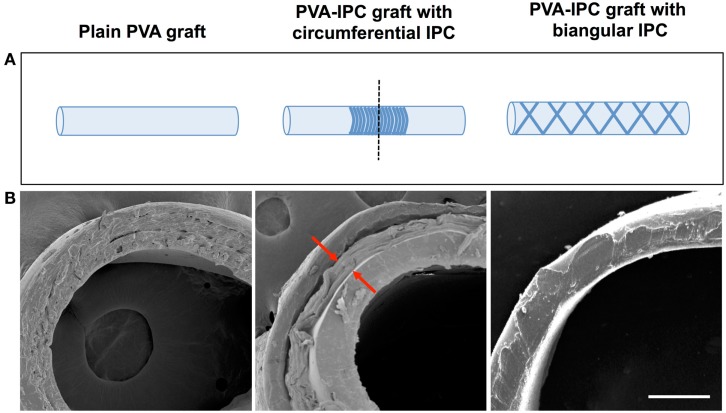
**PVA–IPC composite grafts**. **(A)** Schematic diagram of composite grafts with circumferential alignment and biangular alignment of IPC fibers. Dashed line shows cross-section area imaged with SEM. **(B)** SEM image showing the cross-section of different types of PVA–IPC composite grafts compared with plain PVA graft. Scale bar = 200 μm.

While PVA–IPC grafts with circumferential IPC fibers (Circumferential grafts) showed a marked layer of IPC fibers in between the inner and outer layers of PVA, composite grafts with biangular IPC fibers (Biangular grafts) showed continuity between the PVA layers (Figure 4). Mechanical characterization of the Circumferential and Biangular grafts showed distinct changes in properties depending on the IPC fiber orientation. Circumferential grafts had significantly decreased Young’s moduli, markedly reduced compliance and burst pressure when compared with plain PVA grafts (Table 4). Expectedly, we observed an increased wall thickness in the area of the graft with IPC fibers. Nonetheless, Circumferential grafts closely approximated the Young’s modulus and compliance of rabbit femoral artery. Meanwhile, Biangular grafts showed an opposite effect in increasing Young’s modulus and minimal changes in compliance when compared to plain PVA grafts (Table 5). Burst pressure and wall thickness of Biangular grafts were also notably reduced in comparison with plain PVA grafts.

**Table 4:** **Mechanical properties of PVA–IPC composite grafts with circumferential IPC fibers**.

Scaffold type	Internal diameter (μm)	Wall thickness (μm)	Young’s modulus (kPa)	Compliance (%)	Burst pressure (mmHg)
Plain PVA	1150 ± 72.93	312.7 ± 42.54	562.9 ± 144.2*	8.171 ± 3.463	492.2 ± 66.88
Circumferential composite graft	1072 ± 73.85	598.4 ± 120.9 (part with IPC fiber)	125.7 ± 37.78*	4.810 ± 5.050	269.4 ± 134.1
Rabbit femoral artery	938 ± 201	350	230	5.9 ± 0.5	2031–4225

*Denotes statistical significance between composite scaffold and plain PVA graft.

**Table 5:** **Mechanical properties of PVA–IPC composite graft with biangular IPC fibers**.

Scaffold type	Internal diameter (μm)	Wall thickness (μm)	Young’s modulus (kPa)	Compliance (%)	Burst pressure (mmHg)
Plain PVA	2690 ± 101.2	375.8 ± 79.6*	352.3 ± 32.59*	4.88 ± 0.24	310.9
Biangular composite graft	1963 ± 76.86	311.3 ± 29.0*	830.5 ± 116.8*	5.23 ± 2.8	202.6 ± 101.9

*Denotes statistical significance between composite scaffold and plain PVA graft.

### Controlled release from PVA–IPC composite grafts

Further examination of biomolecule permeability was performed using PVA–IPC composite grafts that were incorporated with molecules smaller than BSA. The incorporation efficiency of various biomolecules in PVA–IPC composite grafts were optimized by varying the ratio between alginate, chitosan, and heparin (Table 2).

**Figure 5:**
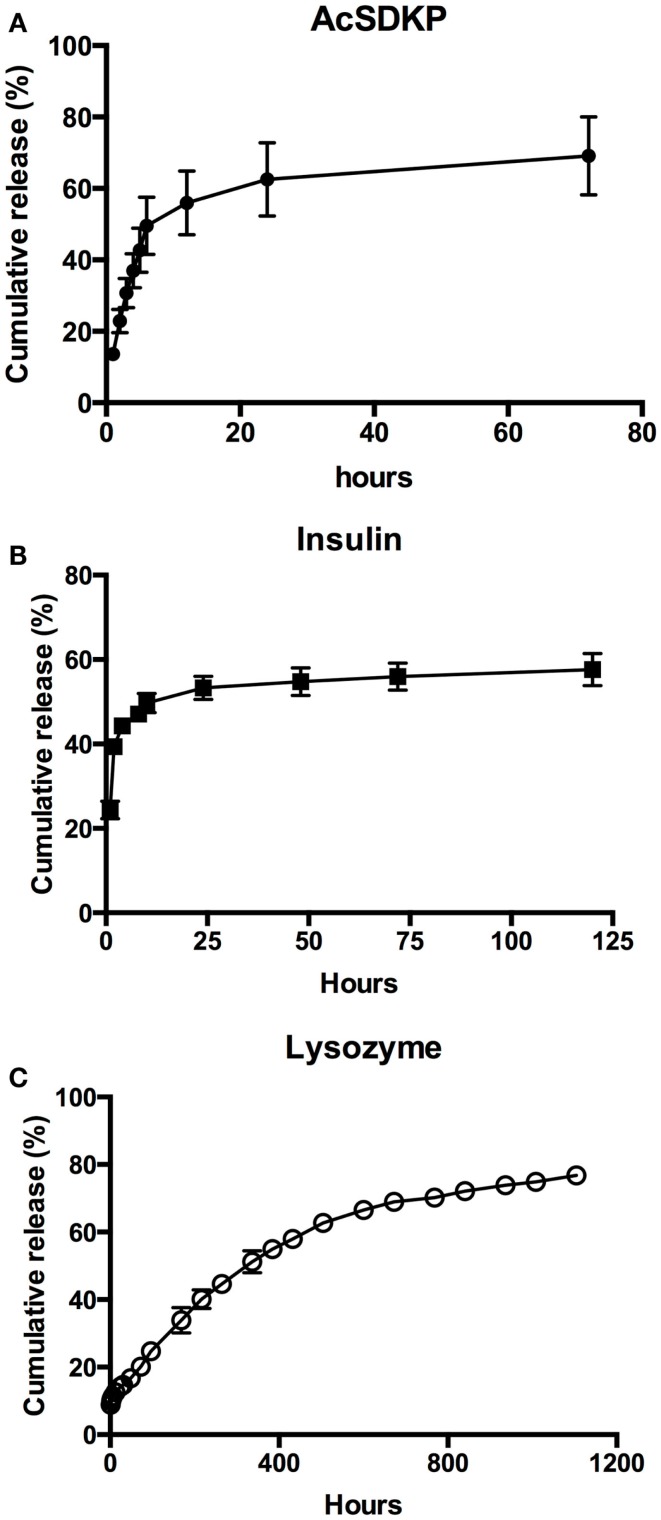
**Controlled release of biomolecules through PVA–IPC composite grafts**. Cumulative release profile of **(A)** AcSDKP, **(B)** insulin, and **(C)** lysozyme from PVA–IPC composite grafts.

AcSDKP release showed burst release until 12 h, with diminishing release rates that began at 24 h after immersion in release medium (Figure 5A). Insulin showed a similar release profile, with a very rapid release within the first 4 h (Figure 5B). Maximum release of 58% insulin was achieved after 24 h. In contrast to AcSDKP, cumulative lysozyme release was nearly linear and with minimal burst release at early timepoints (Figure 5C). However, there was a noted decrease in release rate from 284 h (16 days) timepoint, where the release profile started to show a diminishing rate of release. Maximum lysozyme release of 77% was reached in 92 days. Apparent permeability coefficients calculated from PVA–IPC grafts matched the release profiles observed (Table 6). Modifying the number of outer layers of PVA did not show any change in release profile or maximum cumulative release achieved for lysozyme and BSA (Figure S2 in Supplementary Material).

**Table 6:** **Apparent permeability coefficient of hydrophilic biomolecules calculated from controlled release experiments using PVA–IPC composite grafts**.

Biomolecule	Apparent permeability coefficient (10^−7^ cm/s)
AcSDKP peptide	2.18
Insulin	0.529
PEG-QK	0.43
Lysozyme	0.218
VEGF	0.162

### Controlled release of bioactive angiogenic molecules VEGF and PEG-QK

**Figure 6:**
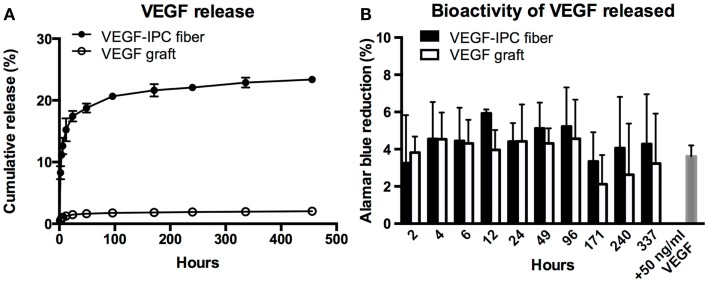
**Controlled release of VEGF from PVA–IPC composite graft with circumferential IPC fibers**. **(A)** Cumulative release profile of VEGF from VEGF composite grafts compared with standalone VEGF-IPC fibers. **(B)** Bioactivity of released VEGF measured by Alamar blue percent reduction.

Thereafter, we demonstrated the use of the PVA–IPC composite grafts with circumferential IPC fibers for the controlled release of angiogenic growth factor VEGF (VEGF graft). Since the molecular weight of VEGF was less than BSA, we postulated that VEGF would be permeable through the composite grafts. Overall, VEGF release profile was similar for both standalone IPC fibers loaded with VEGF (VEGF–IPC fibers) and VEGF grafts. Standalone IPC fibers achieved a maximum cumulative release of 23.40 ± 0.51% yet only a maximum release of 2.03 ± 0.07% VEGF was observed from VEGF grafts after 19 days (Figure 6A). Furthermore, release from VEGF graft achieved a release plateau starting from 171 h (7.1 days).

The bioactivity of released VEGF was also assessed by monitoring HUVEC metabolic activity using Alamar blue assay (Figure 6B). Alamar blue reduction levels were statistically similar for VEGF released from graft or standalone IPC fibers. HUVEC metabolic activity using release media from both scaffolds markedly decreased at 171 h, which marked the plateau of VEGF release. It was notable that the Alamar blue reduction levels were statistically similar to what was induced by supplemented 50 ng/ml VEGF. This indicates that there is good preservation of VEGF bioactivity in both IPC and PVA–IPC composite scaffolds.

VEGF release profile implied its limited diffusion through PVA despite having a molecular weight lower than BSA. We then aimed to use an angiogenic molecule that can mimic the charge and molecular weight of lysozyme (Table 1). QK peptide, an angiogenic molecule that mimics the bioactive helix of VEGF (Finetti et al., 2012) was PEGylated to increase its molecular weight to the range of the lysozyme molecular weight (Supplementary Material). PEG conjugation of QK did not change its bioactivity (Figure S1 in Supplementary Material) and was incorporated in PVA–IPC composite grafts (PEG-QK graft) to study its release behavior.

**Figure 7:**
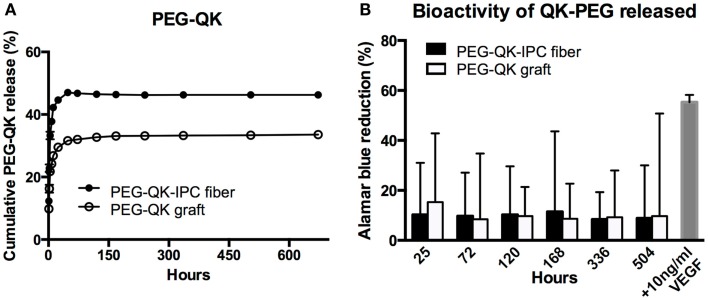
**Controlled release of PEG-QK from PVA–IPC composite graft with biangular IPC fibers**. **(A)** Cumulative release profile of PEG-QK from PEG-QK composite graft compared with standalone PEG-QK-IPC fibers. **(B)** Bioactivity of released PEG-QK measured by Alamar blue percent reduction.

In general, the release profile of PEG-QK from both PEG-QK-IPC fibers and PEG-QK grafts were similar (Figure 7A). For both types of scaffolds, an initial rapid release was followed by a diminished rate of release. Similar to VEGF release profile, a higher maximum PEG-QK release was achieved from standalone IPC fibers at 46% in comparison to PEG-QK grafts, achieving maximum of 33% in 5 days.

Release medium from both PEG-QK-IPC fibers and PEG-QK graft had a positive effect on HUVEC metabolic activity, signifying the bioactivity of PEG-QK (Figure 7B). Compared with supplemented VEGF, the bioactivity of released PEG-QK was found to be markedly lower than 10 ng/ml VEGF. Nonetheless, the observed bioactivity of PEG-QK was consistent through all timepoints and showed no significant differences between media from either PEG-QK-IPC fibers or PEG-QK grafts.

### Proof-of-concept: Feasibility of PVA–IPC composite graft implantation in rabbit model

**Figure 8:**
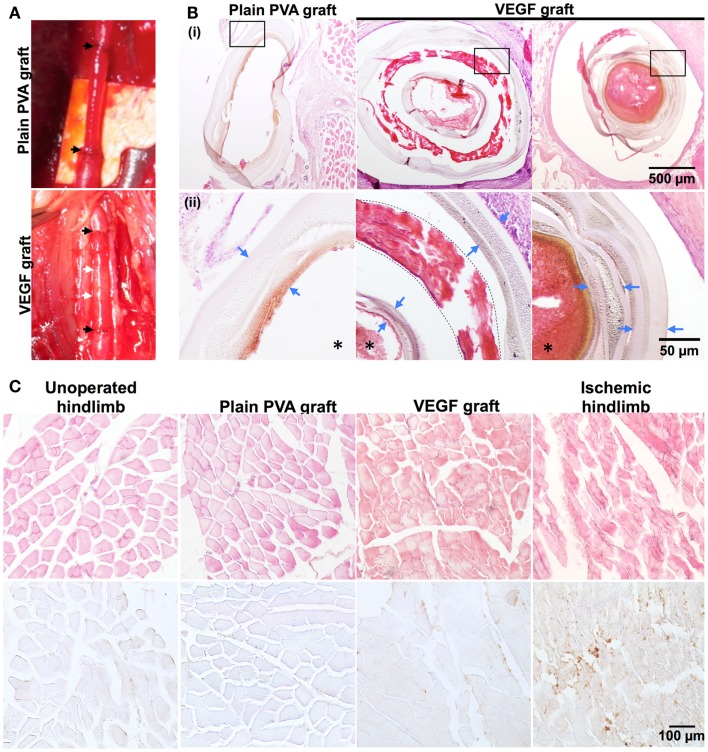
**Proof-of-concept implantation of PVA–IPC composite graft in rabbit femoral artery**. **(A)** Representative PVA graft and VEGF graft implanted in rabbit femoral artery. Black arrows represent anastomosis of graft to femoral artery. White arrow denotes location of circumferential IPC fibers. **(B)** Hematoxylin and eosin (H&E) stain against plain PVA graft and VEGF graft. (i) Shows overview of grafts (10X). Box indicates magnified area shown in (ii) (20X). Blue arrows denote PVA layers, dashed line denotes the IPC fiber layer in the VEGF grafts and *denotes the lumen of the grafts. **(C)** H&E stain and IHC stain against Mac-387 on various hindlimb tissues surrounding the graft (20X). Unoperated hindlimb and ischemic hindlimb were used as negative and positive controls, respectively. Brown color denotes positive stain for Mac-387.

Afterwards, VEGF grafts were anastomosed to the rabbit femoral artery. VEGF grafts were chosen because of the higher bioactivity of VEGF retained in comparison to PEG-QK. Both the VEGF and PVA grafts were easily anastomosed to the rabbit femoral artery (Figure 8A), resulting in maximum ischemia time of only 1 h and 30 min. Surgical sites appeared normal and without edema throughout recovery. All animal subjects showed alertness, responsiveness, normal appearance, and mobility at 28 days. No systemic abnormalities were observed for any of the rabbit subjects.

Histological analysis of the PVA grafts after 28 days of implantation showed that both VEGF grafts and plain PVA grafts did not show any signs of degradation (Figure 8B). However, stenosis of the VEGF graft was observed (Figure 8B). A thin layer of connective tissue or capsule was seen around the VEGF grafts, in contrast to PVA grafts (Figure S3 in Supplementary Material). Standard H&E stains of hindlimb muscles show rounded and bundled muscle fibers around PVA and VEGF grafts. These hindlimb muscles closely resembled the normal physiological hindlimb muscles in the unoperated control. Macrophage infiltration was also assessed through histology to determine acute inflammation against plain PVA and VEGF grafts. Mac-387 staining was similar in hindlimb muscles surrounding PVA graft and VEGF graft (Figure 8C). In addition, hindlimb muscles from both PVA and VEGF grafts showed Mac-387 stain density similar to an unoperated hindlimb. Meanwhile, ischemic muscles showed angular muscle fibers with large bundle separation and a higher density of Mac-387 positive stain compared with the unoperated control. Connective tissues surrounding PVA and VEGF grafts also showed similarities in staining against Mac-387 (Figure S3 in Supplementary Material).

## Discussion

Therapeutic protein delivery, local concentration, and bioactivity must be strictly controlled to induce stable and therapeutic effects *in vivo* (Lee et al., 2011). For example, the lack of clinical benefit of VEGF in clinical trials is caused by indiscriminate growth factor delivery (Rajagopalan et al., 2003). Despite this critical need, many bioengineered platforms for hydrophilic protein delivery are inefficient and incapable of retaining bioactivity. Moreover, this leads to a large amount of protein loss that can be expensive and wasteful. IPC fibers are easily fabricated in ambient and aqueous conditions, leading to efficient biomolecule incorporation and high biomolecule activity. However, IPC fibers are limited in therapeutic application due to its weak mechanical properties. PVA cross-linked with food-grade STMP (Andersen, 2001) supplies a good scaffold for controlled biomolecule delivery due to its desirable mechanical (Chaouat et al., 2008) and biocompatible (Ino et al., 2013) characteristics through a simplistic preparation in aqueous and ambient conditions.

As a possible solution, we combined PVA and IPC fibers to create bioinert, mechanically-strong composite scaffolds for controlled delivery of biomolecules. We observed that PVA showed size-dependent permeability toward biomolecules from the transwell experiments. The large molecule BSA diffused poorly through PVA films while the relatively smaller molecule lysozyme equilibrated across the PVA membrane. Similarly, we observed the same relationship for biomolecule movement through PVA–IPC composite grafts, which was used to demonstrate the potential application of the composite scaffold. Low molecular weight AcSDKP (Wilkins et al., 1999) and insulin showed fast release within 24 h, whereas lysozyme showed a more sustained long term release. Our results are analogous with those of Matsuyama et al. (1997), who observed decreased diffusion of large molecules because of the small mesh size of the glutaraldehyde-cross-linked PVA hydrogel. In contrast, our PVA is cross-linked by STMP, thus creating a hydrogel with physical and chemical characteristics that differentiates it from glutaraldehyde-cross-linked PVA. For instance, our PVA putatively contains negatively charged phosphate linkages (Leone et al., 2010; Ino et al., 2013) that may cause electrostatic repulsion against negatively charged biomolecules such as BSA and insulin. This agrees with Shalviri et al.’s (2010) work on the permeability of negatively charged polysaccharide membranes to different molecules. The group showed that molecules of opposite charge to the polysaccharide membrane had a nearly fivefold increase in permeability compared to molecules of similar molecular weight but possessing the same charge polarity as the membrane. Hydrophilicity may also be a determining factor in the movement of molecules through the highly hydrophilic PVA hydrogel network.

Though VEGF had a molecular weight lower than BSA, we still observed non-optimal diffusion of this growth factor through PVA–IPC grafts. We then sought to use an angiogenic peptide that had a molecular weight similar to lysozyme. PEG-QK peptide showed a similar release profile to VEGF, albeit at a higher cumulative release percentage. While the rate of release remained largely the same for either VEGF or PEG-QK, the cumulative release from standalone IPC fibers always exceeded that of the composite scaffold. Based on our permeability experiments and the similarity of release profiles from both IPC fibers and composite scaffold, we postulate that the PVA may be the limiting step in rate of biomolecule release from the composite scaffolds. Moreover, our results strongly suggest the release from both IPC and PVA–IPC scaffolds can be determined by Fickian diffusion, which is characterized by a diminishing release rate over time (Ritger and Peppas, 1987). The impermeable PVA barrier in PVA–IPC scaffolds further controls release rate, as governed by biomolecule weight and hydrodynamic size (Matsuyama et al., 1997). This is further supported by the contrast in release profile between lysozyme and PEG-QK, (Jevsevar and Kunstelj, 2012; Stiehl et al., 2013) which have similar molecular weights but marked difference in hydrodynamic radius. In contrast, our previous study demonstrated near-linear release of BSA from a composite scaffold made from IPC fibers embedded in porous STMP-cross-linked polysaccharide hydrogel (Cutiongco et al., 2014). Thus, it may be advantageous to change PVA permeability through addition of non-hazardous porogens such as sodium chloride salts. Changing the crystallinity of PVA by using different acetylated and hydrated species of PVA molecules can also change biomolecule movement (Nugent and Higginbotham, 2007).

Aside from the significance of precise temporal release in growth factor therapy, it is necessary to deliver bioactive growth factors to produce a therapeutic effect. We noted the bioactivity of both VEGF and PEG-QK despite the alkaline environment to crosslink the PVA. Notably, there were no significant differences in Alamar blue reduction between IPC fibers and PVA–IPC grafts at any timepoint. In comparison with supplemented VEGF, our results indicate that there is retention of bioactivity when using PVA–IPC scaffolds. Overall, the aqueous and ambient conditions used for PVA–IPC scaffold fabrication retained the ideal IPC fiber property of protecting growth factor activity. Coupled with high biomolecule incorporation efficiency, PVA–IPC scaffolds are advantageous for therapeutic angiogenesis with significantly lower loss of expensive growth factor and reduced manufacturing cost.

While the most important function of IPC fibers is the temporal control of biologics delivery, it must also be noted that the flexibility of manipulating IPC fibers allows control of its geometry, orientation, and incorporation into any type of scaffold. Specifically, IPC fibers were molded while being drawn from the polyelectrolyte solution. With this method, we were able to incorporate IPC fibers onto PVA scaffolds with two different orientations. In both circumferential and bidirectional angular orientations, IPC fibers were integrated in between PVA layers without hindering crosslinks between the PVA layers. The versatility of dictating both IPC fiber geometry coupled with PVA macromolding permits the use of the composite scaffold for other tissue engineering applications such as wound dressing or peripheral nerve graft.

In addition, the superb mechanical strength of PVA–IPC composite scaffolds is desirable for *in vivo* applications, such as a bioactive vascular graft with growth factor or drug delivery. It was previously demonstrated that PVA mechanical properties can be easily tuned to approach native arterial characteristics (Chaouat et al., 2008). While plain PVA grafts exhibited mechanical strength, the addition IPC fibers were able to further tweak the mechanical properties to more closely approximate the rabbit femoral artery. We observed that the orientation of IPC fiber alignment produced different effects on mechanical characteristics. Concentration of IPC fibers in the circumferential arrangement may create an area of discontinuity that decreases elastic modulus, compliance, and burst pressure. Biangular grafts, on the other hand, had more widely and uniformly distributed IPC fibers that may be more beneficial in spreading mechanical load thus leading to increased elastic modulus while retaining radial compliance. Nonetheless, the mechanical characteristics of circumferential grafts that closely approximated the rabbit femoral artery facilitated anastomosis and reduced vessel injury, and decreasing surgery time and post-operative complications.

*In vivo* implantation of PVA or VEGF grafts for 28 days did not result in drastic or macroscopic change in graft structure. The lack of clinical (e.g., edema) or histological manifestations of local inflammation and the similarities in capsule formation demonstrated that PVA–IPC composite grafts have similar bioinertness as PVA grafts. Nonetheless, the luminal stenoses of the grafts due to possible thrombosis or neointimal growth imply that further fine-tuning of the mechanical and biomolecule release properties of the VEGF grafts could be performed.

Dual release of growth factors is important for synergistically inducing angiogenesis (Hao et al., 2007). Recently, Han et al. (2013) showed the dual delivery of VEGF and platelet derived growth factor from electrospun vascular grafts. Despite the impressive release profile of growth factors, they only demonstrated maximum 20% incorporation efficiency and lack of radial compliance. Use of IPC fibers provide a more versatile method for efficient multiple growth factor incorporation that is not limited to heparin-binding factors. IPC fiber technique is not limited to growth factors and may also be used for cell, DNA, or microparticle delivery for therapeutic angiogenesis (Yim et al., 2006; Tai et al., 2010; Yow et al., 2011). Most notably, our PVA–IPC scaffold can be fabricated in aqueous and ambient conditions that aid the preservation of growth factor bioactivity thus, increasing its application in therapy.

In summary, we have shown the potential of PVA–IPC composite scaffolds as a new platform for controlled hydrophilic biomolecule delivery. Firstly, we showed the size-dependent permeability of hydrophilic molecules through PVA. Secondly, we showed that PVA–IPC composite scaffolds showed excellent mechanical properties that were modulated by IPC fiber alignment. Thirdly, PVA–IPC composite tubular scaffold was fabricated to demonstrate its application as a vascular graft. Fourthly, controlled release of hydrophilic molecules through PVA–IPC composite scaffolds were also demonstrated through a systematic study of various model molecules, where lysozyme showed near-linear release for 30 days. Significantly, PVA–IPC grafts showed retention of angiogenic growth factor bioactivity. Finally, PVA–IPC grafts were implantable in a rabbit model and showed bioinertness, eliciting a similar local inflammatory response as PVA grafts. PVA–IPC fiber composite scaffolds represent an excellent platform growth factor delivery for various applications in soft tissue engineering.

## Author Contributions

All authors contributed to the design of the experiments and approval of the manuscript. Marie Francene A. Cutiongco, Royden K. T. Choo, Nathaniel J. X. Shen, Ervi Sju, and Amanda W. L. Choo performed the controlled release experiments. Royden K. T. Choo performed the permeability experiments. Bryan M. X. Chua contributed to the mechanical testing of the grafts. Marie Francene A. Cutiongco performed histological analysis. Catherine Le Visage and Evelyn K. F. Yim provided scientific expertise. Marie Francene A. Cutiongco wrote the manuscript. Evelyn K. F. Yim edited and gave final approval for submission of the manuscript.

## Conflict of Interest Statement

The authors declare that the research was conducted in the absence of any commercial or financial relationships that could be construed as a potential conflict of interest.

## Supplementary Material

The Supplementary Material for this article can be found online at http://www.frontiersin.org/Journal/10.3389/fbioe.2015.00003/abstract
