# Supplementary material for: Composite Scaffold of Poly(Vinyl Alcohol) and Interfacial Polyelectrolyte Complexation Fibers for Controlled Biomolecule Delivery
Source: Front Bioeng Biotechnol. 2015 Feb 3;3:3. doi: 10.3389/fbioe.2015.00003 (PMC4315105; doi:10.3389/fbioe.2015.00003)
Supplement: Supplementary file 1 [file Data_Sheet_1.DOCX]

***Supplementary Material***

**Composite scaffold of poly(vinyl alcohol) and interfacial polyelectrolyte complexation fibers for controlled biomolecule delivery**

**Marie Francene A. Cutiongco^1^, Royden K. T. Choo^1^, Nathaniel J. X. Shen^1^, Bryan M. X. Chua^1^, Ervi Sju^1^, Amanda W. L. Choo^1^, Catherine Le Visage^2,3^ and Evelyn K. F. Yim^1,4,5^**

^1^Department of Biomedical Engineering, National University of Singapore, Singapore

^2^INSERM, U698 Cardiovasvular Bioengineering, Paris, France

^3^INSERM, U791 Center for OsteoArticular and Dental Tissue Engineering, Nantes, France

^4^Mechanobiology Institute, Singapore, National University of Singapore, Singapore

^5^Yong Loo Lin School of Medicine, Department of Surgery, National University of Singapore, Singapore

*** Correspondence:**

Dr. Evelyn KF Yim

Department of Biomedical Engineering

National University of Singapore

Block EA, #03-12

9 Engineering Drive 1

117575 Singapore

eyim@nus.edu.sg

1. **Supplementary Data**

## PEGylation of QK peptide

QK (WQELYQLKY, 1270.44Da, 98.7% purity, GenScript) was conjugated to Biotin-PEG-NHS (NANOCS, 10kDa, >95% purity, substitution purity >80%) and MPEG-Succinimidyl-NHS (NANOCS, 10kDa, >95% purity, substitution purity >90%) to PEGylated QK conjugates (11.27kDa). Selective biotin-labeling of N-terminal α-amino group was performed. PEG-QK was purified using Amicon Ultra-4 Centrifugal Filter Unit with Ultracel-10 membrane (molecular weight cut off 10 kDa) to remove unreacted QK peptide. The PEG-QK conjugate was kept at -20°C until future use.

## Determining bioactivity of PEG-QK

Matrigel and Alamar blue cell proliferation assays were conducted after conjugation to determine biological activity of PEG-QK conjugates. HUVEC were seeded onto 96 well plates at 2000 cells/well. After 18 hours, HUVEC were grown in serum-reduced media (SRM) comprised of endothelial cell basal medium (EBM, Lonza) with 1% fetal bovine serum (FBS), ascorbic acid, hydrocortisone and gentamicin. After 24 hours of growth, SRM was replaced with 150 μl of SRM with PEG-QK, QK, PEG or recombinant human VEGF (Life Technologies). Molar concentrations of each growth factor component except VEGF was kept constant at 10^-6^ M. VEGF was added at 10^-12^ M, since VEGF had twice as high activity as QK (Santulli et al., 2009). Cells were then incubated at standard cell culture conditions. Alamar Blue assay was carried out on days 1, 3, 5 and 7 to determine cell metabolic activity. Figure S1A shows statistically comparable levels of Alamar blue reduction induced by PEG-QK, QK and VEGF at all timepoints tested.

Matrigel assay was also conducted to observe in vitro tubular network formation. The extent of network formation was indicative of angiogenic effect of PEG-QK. Growth Factor Reduced Matrigel (BD Bioscience, 50 µl) was added to 96-well plate. The plate was incubated at 37°C for 1 hour before 15,000 cells resuspended in SRM with growth factor components were seeded into each well. HUVEC were allowed to grow at standard incubation conditions before vessel formation was noted at 24 hours. Figure S1B shows comparable level of tubular formation in Matrigel between PEG-QK and VEGF.

1. **Supplementary Figures and Tables**

**Figure S1. Bioactivity of PEG-QK conjugate.** **(A)** Cell activity, as measured by Alamar blue percent reduction, of HUVEC cultured with PEG-QK conjugates in serum reduced medium. **(B)** In vitro angiogenic capacity of HUVEC cultured with PEG-QK conjugates in serum reduced medium. Images were taken 24 hours post-seeding. Scale bar – 500 μm.

**Figure S2. Permeability of biomolecules through PVA films with 3 outer layers.** Transwell assay was used to assess permeability of **(A)** lysozyme and **(B)** BSA through PVA film with 3 layers. Top chamber denotes chamber anterior to the transwell insert, where the biomolecules were added. Bottom chamber denotes the chamber enclosed by the tissue culture well plate and posterior to the transwell insert, where biomolecules diffuse through.

**Figure S3. Capsule formation around PVA-IPC and PVA grafts. (A)** H&E showing the connective tissue (capsule) formed around representative PVA and VEGF grafts implanted in rabbit femoral artery (20X). **(B)** IHC stain against macrophage marker Mac-387 on various hindlimb tissues surrounding the graft (20x). Unoperated hindlimb and ischemic hindlimb were used as negative and positive controls, respectively, for Mac-387 staining.
