# Supplementary figures and images for: Composite Scaffold of Poly(Vinyl Alcohol) and Interfacial Polyelectrolyte Complexation Fibers for Controlled Biomolecule Delivery
Source: Front Bioeng Biotechnol. 2015 Feb 3;3:3. doi: 10.3389/fbioe.2015.00003 (PMC4315105; doi:10.3389/fbioe.2015.00003)

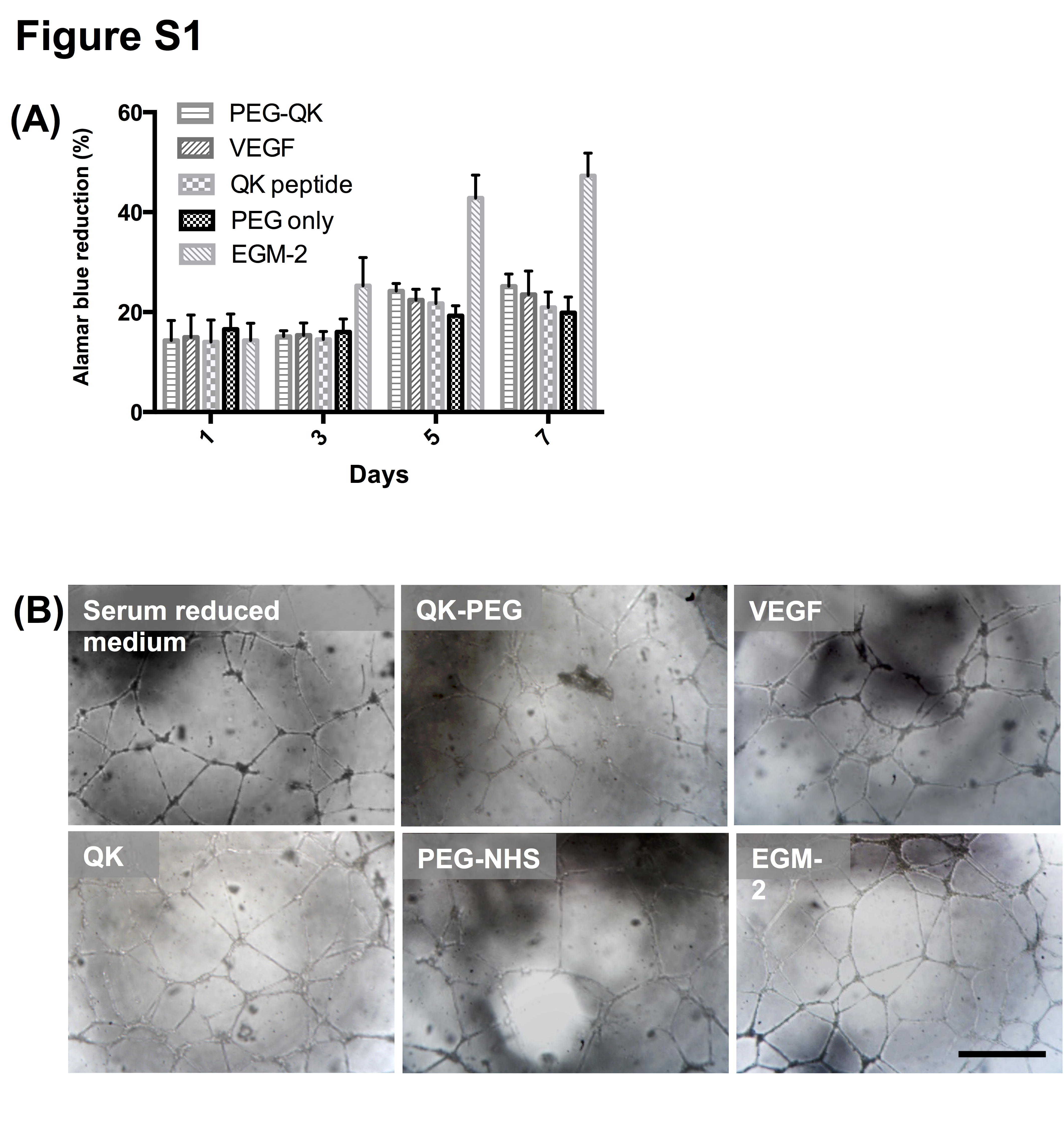

Supplement: Supplementary file 2 [file Image_1.TIFF]

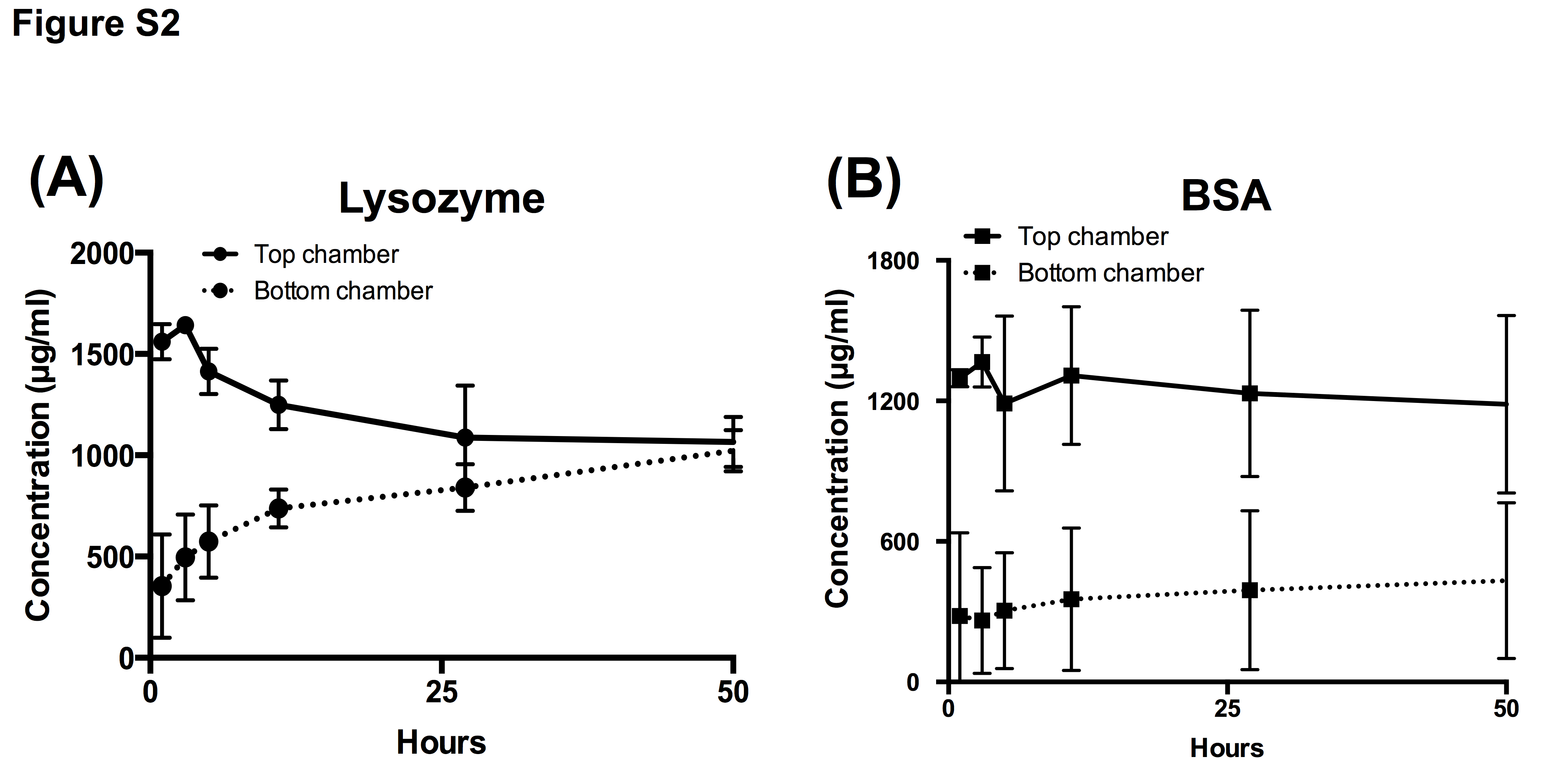

Supplement: Supplementary file 3 [file Image_2.TIFF]

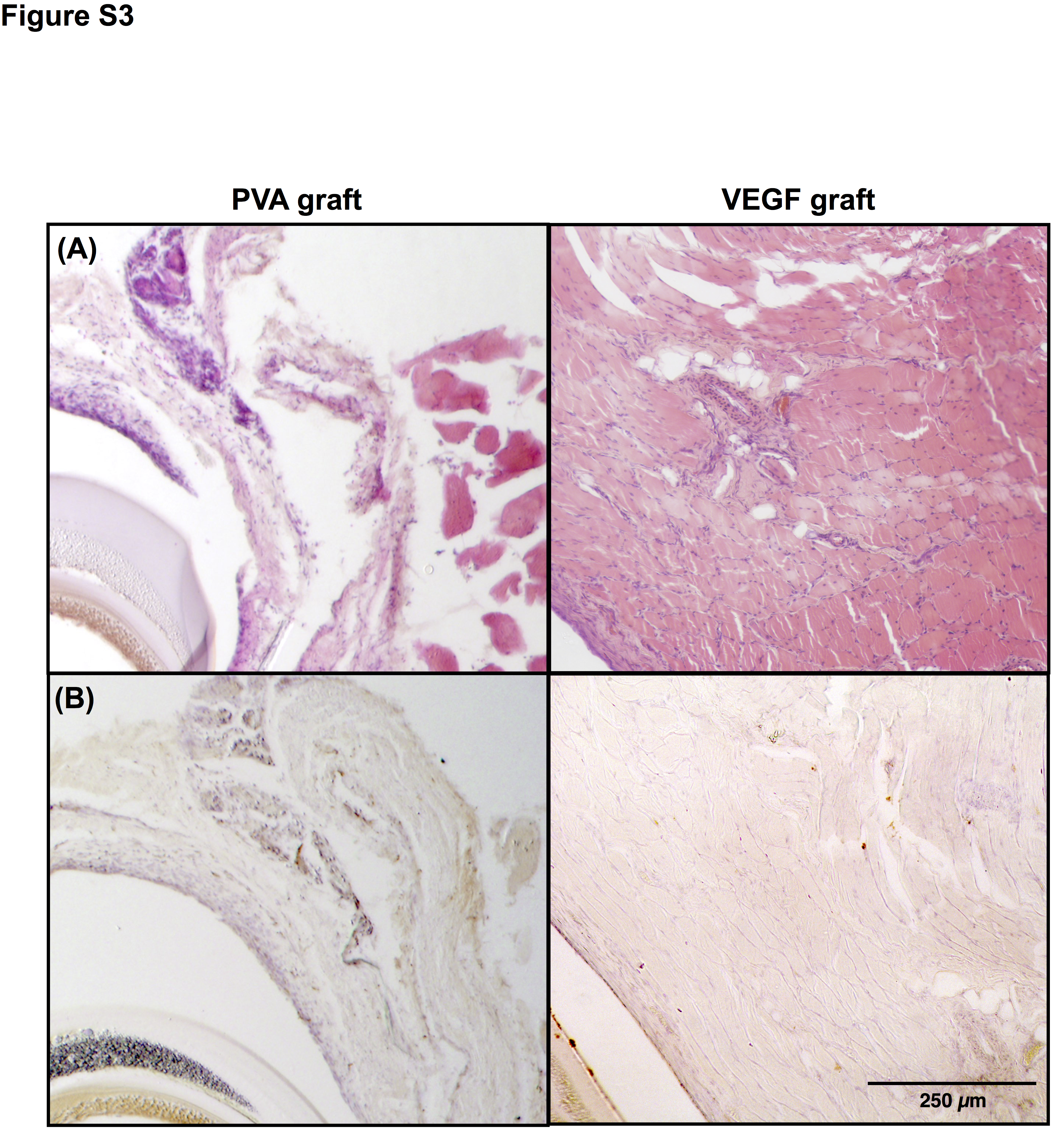

Supplement: Supplementary file 4 [file Image_3.TIFF]
